# Thoracic joint abnormalities and impaired thoracic motion in idiopathic pleuroparenchymal fibroelastosis: an exploratory study using inspiratory and expiratory CT

**DOI:** 10.1186/s12890-026-04379-9

**Published:** 2026-06-12

**Authors:** Takayuki Takimoto, Eiji Sugimoto, Tomoko Kagawa, Kazuomi Sugamoto, Toru Arai

**Affiliations:** 1 Department of Respiratory Medicine, NHO Kinki Chuo Chest Medical Center, 1180 Nagasone-cho, Kita-ku, Sakai City, Osaka 591-8555 Japan; 2 Clinical Research Center, NHO Kinki Chuo Chest Medical Center, 1180 Nagasone-cho, Kita-ku, Sakai City, 591-8555 Osaka Japan; 3 Graduate School of Engineering, Osaka University, 2-1 Yamada-oka, Suita, 565-0871 Osaka Japan

**Keywords:** Thoracic motion, Interstitial lung disease, Pleuropulmonary parenchymal fibroelastosis, Thoracic cage joints, Three dimensional-computed tomography

## Abstract

**Background:**

Idiopathic pleuroparenchymal fibroelastosis (IPPFE) is a rare interstitial lung disease characterized by restricted ventilation and upper-lobe fibroelastosis. Historically, ankylosing spondylitis has been implicated in upper-lobe fibrosis and ventilatory impairment due to chest wall restriction, and impaired thoracic motion has recently been appreciated in IPPFE. We hypothesized that thoracic joint abnormalities (costovertebral, manubriosternal, and sternocostal joints) contribute to the impaired thoracic motion in patients with IPPFE.

**Methods:**

Seventeen patients with IPPFE and 15 non-PPFE interstitial lung disease were retrospectively investigated. Thoracic joint abnormalities were assessed as abnormal calcifications on inspiratory CT images. Thoracic motion was evaluated by the average moving distance of four anterior thoracic landmarks between inspiratory and expiratory CT images. Thoracic motion ratio (TMR) was defined as the ratio of the measured average moving distance to the cube root of the predicted vital capacity. The ratio of the lung volume change (LVCR) was calculated from inspiratory and expiratory CT images. Associations between TMR, LVCR, and pulmonary function test parameters (PFT) parameters were assessed.

**Results:**

Thoracic joint abnormalities were observed in 14 of 17 IPPFE patients, mainly at sternocostal joints. Impaired thoracic motion, defined by the thoracic motion ratio less than half of the normal value, was observed in 9 of 17 IPPFE patients, but in only 1 of 15 non-PPFE patients. All 9 IPPFE patients with impaired thoracic motion exhibited joint abnormalities. The correlation between TMR and LVCR was stronger in the IPPFE group than in the non-PPFE group.

**Conclusion:**

Thoracic joint abnormalities associated with impaired motion are frequently observed in IPPFE patients. These abnormalities may restrict thoracic movement and lead to the development of IPPFE.

## Introduction

Pleuroparenchymal fibroelastosis (PPFE) is a rare form of interstitial lung disease (ILD) characterized radiologically by dense subpleural fibrosis and upper lobe volume loss [1–3], which is associated with restrictive ventilatory impairment. Idiopathic PPFE (IPPFE) was included as a rare form of idiopathic interstitial pneumonia in the official American Thoracic Society (ATS) /European Respiratory Society (ERS) 2013 classification [3]. In the 2025 ERS/ATS international multidisciplinary classification, PPFE is retained as a distinct interstitial pneumonia pattern within a broader, morphology-based classification that encompasses both idiopathic and secondary ILD causes [4].

IPPFE is associated with progressive restrictive ventilatory impairment, the underlying pathogenesis of which remains poorly understood. Secondary forms of PPFE have been reported in association with collagen vascular diseases, hematopoietic stem cell transplantation, infections, radiation therapy, and pharmacologic agents [5]. Conditions such as ankylosing spondylitis (AS) [6, 7], neuromuscular disorders (e.g., amyotrophic lateral sclerosis; ALS) [8], and post-thoracotomy changes [9] have been implicated in PPFE-like changes. Although impaired thoracic motion may result from fibrotic volume loss of the upper lobes, those conditions associated with reduced thoracic cage movement [6–9] suggest the possibility that impaired thoracic mobility may precede pleural and parenchymal pathologic change. Impaired thoracic motion in IPPFE has recently been appreciated with dynamic magnetic resonance imaging [9, 10]; however, evidence of thoracic motion in patients with IPPFE remains lacking. In these studies, the measurement methods involved assessing the anteroposterior and/or craniocaudal diameters on a fixed sagittal slice and evaluated deformation of the thoracic cavity rather than motion of the thoracic cage. Therefore, it is impossible to identify the movement of individual thoracic cage structures, such as the sternum and ribs. Mobility of the thoracic cage depends on its skeletal structures, including the ribs, vertebrae, sternum, and associated joints. During respiration, changes in intrathoracic volume are achieved through coordinated alterations in the positional relationships within the thoracic joints. Costovertebral joints (CVJs) consist of two synovial parts that connect the proximal end of the ribs with their corresponding vertebrae. One part is at the head, the other is at the tubercle of the rib, and the line connecting the two parts is a rotation axis of the rib during respiration [11]. In ankylosing spondylitis, where the CVJs are affected, upper-lobe fibrosis and ventilatory impairment secondary to chest wall restriction have been reported [6, 7]. Sternocostal joints (SCJs) are between sternum and the costal cartilages from the first to seventh ribs. The first SCV is synchondrodial and the other are synovial joints where the front-end face of the costal cartilage slides according to the rib rotation [12]. The manubriosternal joint (MSJ) is a symphysis between the sternal manubrium and the sternal body. The angle between the manubrium and the body is called “sternal angle” which changes during respiration [12]. Structural or functional impairment of the thoracic joints, specifically the CVJs, SCJs, and MSJ, may significantly reduce the thoracic motion. In this exploratory study, we aimed to assess thoracic joint abnormalities and thoracic cage motion in patients with IPPFE using end-inspiratory and end-expiratory three-dimensional computed tomography (3D-CT) images. Idiopathic pulmonary fibrosis (IPF) was included as a disease control because it is the most common and well-established ILD with predominant lower-lung involvement, whereas fibrotic hypersensitivity pneumonitis (fHP) was included as a comparator because of its relative upper-lobe involvement.

## Methods

### Study population

Seventeen patients with IPPFE who underwent paired inspiratory and expiratory 3D-CT with a 1-mm slice thickness between May 2021 and July 2025 at the NHO Kinki Chuo Chest Medical Center were retrospectively included. For comparison, 10 patients with IPF and 5 with fHP who underwent inspiratory and expiratory 3D-CT were consecutively selected as controls, starting from the same date (May 2021). IPPFE diagnosis was based on the radiological criteria (probable) proposed by Watanabe [13], whereas IPF [14] and fHP (definite or high confidence) [15] were diagnosed in accordance with the respective ATS/ERS guidelines. Diagnoses were initially made by treating respiratory physicians, and cases without prior formal multidisciplinary discussion were re-reviewed by the investigators according to the same criteria. Those with pleural abnormalities potentially affecting thoracic motility and overt upper lobe fibrotic lesions in IPF were excluded. Clinical and radiological findings were examined. This study was approved by the Ethics Committee of NHO Kinki Chuo Chest Medical Center on December 14th, 2023 (Approval No. Rin-2023-83) and was performed in accordance with the Declaration of Helsinki. The opt-out method was used, and information regarding the study was posted on our website to provide patients with the opportunity to refuse participation.

### Assessment of thoracic cage structure

There is limited information in the literature regarding thoracic joint abnormalities that may impair respiratory movement. A pragmatic semi-quantitative scale was applied for exploratory assessment. Calcification of the joint space and/or surrounding ligaments is readily detectable on 3D-CT images and may impair joint motion. Therefore, we focused on joint calcifications on inspiratory CT images and tentatively defined “joint abnormalities” as follows:

1. Sternocostal joints (SCJs)
  Of the upper seven SCJs, the first SCJ is not synovial, and the synovial cavities of the sixth and seventh SCJs often disappear with age [16, 17]. Therefore, we investigated the second to fifth SCJs. Although it is well known that costal cartilages ossify with age, there is no description of SCJ calcification [17]. Yekeler et al. reported SCJ calcification only at the second SCJ in two of 12 cadavers using multidetector CT (MDCT) [18], suggesting that SCJ calcification is not a common phenomenon. We defined a “calcified SCJ” as the presence of a calcified bridge between the sternal and costal sides of the SCJ observed over two consecutive slices (2 mm) on inspiratory CT images. In addition, we assumed that compensatory movement of the adjacent and contralateral ribs would function if the number of abnormal SCJs is two or fewer. Therefore, we defined “SCJ abnormality” as the presence of three or more calcified SCJs.
2. Manubriosternal joint (MSJ)
  We defined an “MSJ abnormality” as calcification involving more than one-third of the joint width. Although the manubrium and the body of the sternum are originally separate bones connected by the MSJ, complete fusion without finding of osteodegeneration is sometimes observed as a developmental anomaly. Yekeler et al. reported a frequency of 20% among 1,000 patients who underwent MDCT of the chest [18]. In such cases, it is assumed that the movement of the thoracic cage may be optimized just after birth. Therefore, we did not judge complete fusion to represent an abnormal MSJ.
3. Costovertebral joints (CVJs)
  Although deformation of the thoracic vertebral bodies is often observed with age, involvement of the CVJ is rare. We defined “CVJ abnormality” as the presence of calcification in three or more CVJs.

### Assessment of thoracic motion

Image analysis was performed using the 3D Slicer software platform [19]. Because the thoracic vertebral movement is negligible during CT examination at supine posture, we assigned four characteristic points in the anterior part of the thoracic cage and calculated moving vectors by measuring coordinates in inspiratory and expiratory 3D-CT images. Four characteristic points were the top of the manubrium and midpoints of the anterior surface lines of the costochondral joints of the second, forth, and sixth ribs. When the sixth costochondral joint was out of the image, the fifth was measured. When the location of costochondral joint is unclear due to ossification of cartilages, a border point between ossified and non-ossified regions was assigned. The right thorax was selected for analysis because the left thorax is affected by cardiac motion. Because our measurement method involves identifying the 3D coordinates of characteristic points, clear images without motion artifacts are desirable.

**Fig. 1:**
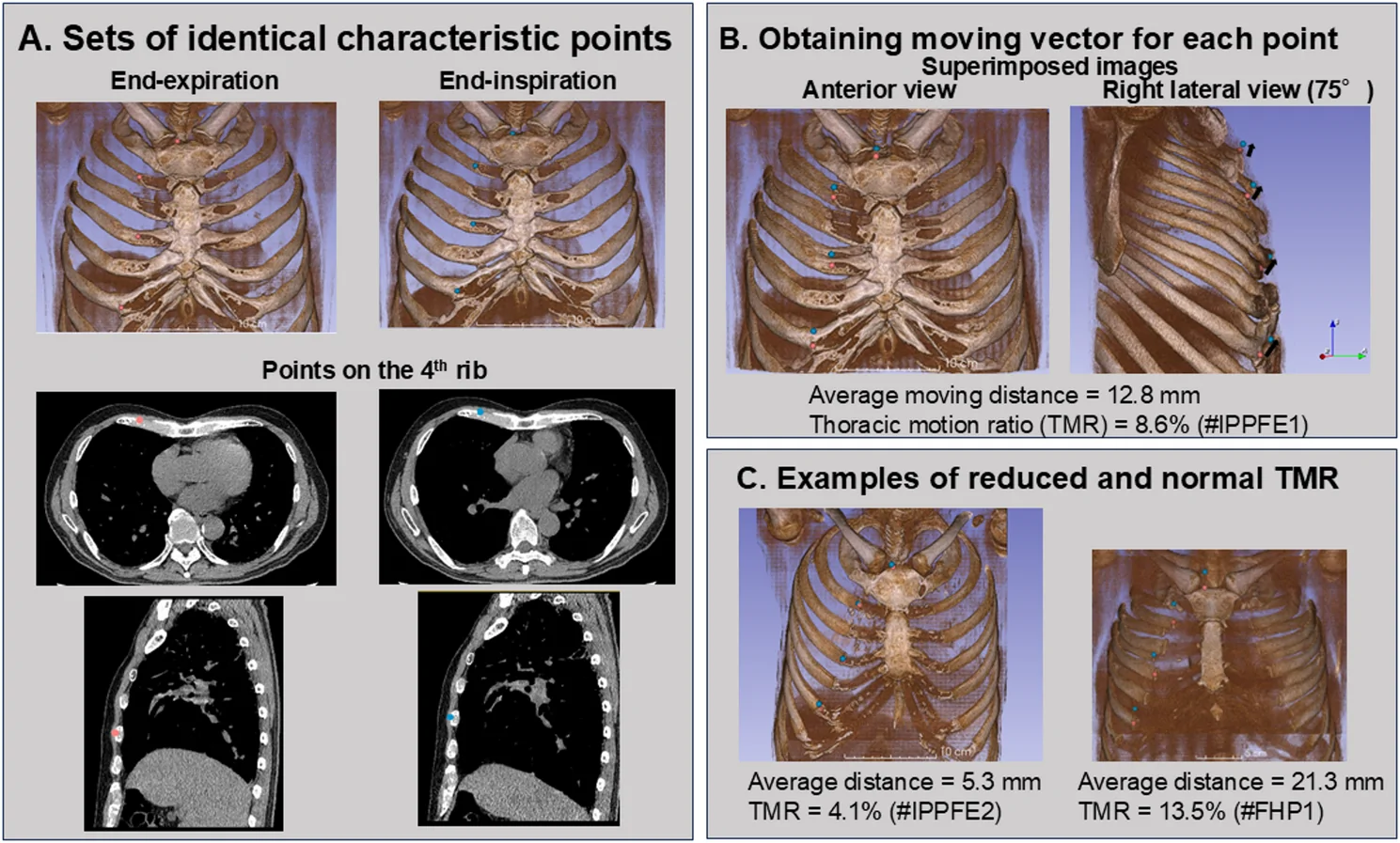
Process for assessing thoracic motion. **A** Four characteristic points were assigned on end-expiratory images (red points), and the corresponding points on inspiratory images (blue points) were identified by matching identical bony structures on axial, coronal, and sagittal slices. **B** A superimposed image of the inspiratory and expiratory thoracic cages illustrates the moving distance of each characteristic point from end-expiration to end-inspiration (#IPPFE1). **C** Representative superimposed images demonstrating severely impaired motion (left, #IPPFE2) and normal motion (right, #FHP1)

Figure 1A shows an example in which identical characteristic points are assigned on expiratory (red points) and inspiratory (blue points) images. Inspiratory and expiratory points were identified by matching identical bony landmarks on axial, coronal, and sagittal slices. Figure 1B shows superimposed images of the inspiratory and expiratory thoracic cages, illustrating how each characteristic point moves from end-expiration to end-inspiration (#IPPFE1). The moving distance (i.e., the length of the movement vector) for each point was calculated from the coordinates of the expiratory and inspiratory points. Figure 1C shows representative cases of severely impaired motion (#IPPFE2) and normal motion (#FHP1), respectively. The average value of the four moving distances was defined as the thoracic moving distance. Because thoracic motion is influenced by sex, age, and height, like vital capacity, the absolute value of thoracic moving distance should be normalized for comparison. Then, we defined “the thoracic motion ratio (TMR)” as the ratio of the thoracic moving distance to the cube root of the predicted vital capacity. In healthy subjects, the mean thoracic displacement is approximately 20 mm [12]. When the predicted lung capacity is 3,500 mL, and the thoracic cavity is assumed to be a cube, the length difference of the cube between inspiratory and expiratory phases is approximated at ∛3,500 = 15.2 cm. The TMR is then calculated as 2.0 / 15.2 = 13.2%. Finally, we set the threshold for impaired thoracic motion at 7%, which corresponds to approximately half of 13.2%, rounded to the nearest integer.

Lung volumes on inspiratory and expiratory CT images were automatically computed using 3D image analysis software, and the ratio of the lung volume change (LVCR) was calculated as (inspiratory volume − expiratory volume) / expiratory volume.

Pulmonary function tests (PFTs) were performed using the gas dilution method with a CHESTAC-8800 or 8900 spirometry system (CHEST M.I., Inc., Tokyo, Japan) to obtain PFT parameters, including vital capacity (VC), residual volume (RV), total lung capacity (TLC), and the diffusing capacity of the lung for carbon monoxide (DLco).

### Statistical analysis

Continuous variables, including PFT parameters (%VC, %RV/TLC, and %DLco), TMR, and LVCR, were compared between the PPFE and non-PPFE groups using the Mann-Whitney U test. Data are presented as medians with interquartile ranges [IQR]. A p-value of < 0.05 was considered statistically significant. Correlations between TMR and LVCR, as well as between TMR and PFT parameters (%VC, %RV/TLC, and %DLco), were assessed using Spearman’s rank correlation coefficient in the IPPFE and non-PPFE groups. Scatter plots with linear regression lines were generated to illustrate these associations.

## Results

### Patients’ characteristics

Patients with asbestosis, bilateral pleural effusion due to atrial fibrillation, or pectus excavatum were excluded. Seventeen patients with IPPFE and 15 non-PPFE patients were included in the study. The IPPFE and non-PPFE groups were similar in age and sex. Body mass index (BMI) and PFTs, including %VC, %RV/TLC, and %DLco reflected the characteristics of IPPFE (Table 1). The PPFE group showed a significantly lower %VC (68.3 [47.90–74.10] vs. 85.9 [73.95–99.55], *p* = 0.007) but a significantly higher %RV/TLC (133.0 [114.70–179.30] vs. 89.1 [80.40–94.85], *p* < 0.001) and %DLco (87.6 [72.55–102.45] vs. 69.7 [60.20–75.75], *p* = 0.023) compared to the non-PPFE group (Table 2).

**Table 1:** Patient characteristics of IPPFE and non-PPFE

						Pulmonary function test			Thoracic joint abnormalities				Thoracic cage motion			
Case	Age,years	Sex	BMI,kg/m^2^	Smoking	Emphysema	VC	RV/TLC	DLco	SCJ	MSJ	CVJ	Total assessment	Average distance,mm	TMR	Impairment	LVCR
				pack-years		%pred	%pred	%pred								
IPPFE																
1	69	M	21.2	38	+	71	114.7	102.9	+			+	12.8	8.6		0.47
2	77	F	14.4	Never		44.7	187	74.3	+	+		+	5.3	4.1	+	0.22
3	70	M	23	10		82	127.7	91.8	+	+		+	7.3	4.8	+	0.48
4	78	M	19	25.5		73.4	133	102	+		+	+	2.9	2.0	+	0.15
5	73	F	13.1	10		35.7	219.3	ND	+			+	6.3	5.0	+	0.20
6	45	M	20.1	Never		73.9	117.6	71.1					21.1	13.4		1.28
7	68	M	17.6	Never		99.7	112.2	108.8	+			+	13.0	8.5		0.48
8	67	F	16.7	Never		61.3	134	74	+			+	22.7	17.2		0.56
9	69	M	17	39		68.3	123.2	110.6			+	+	12.3	8.4		0.38
10	72	M	22.6	Never		103.4	70.9	87.6					29.7	20.1		0.64
11	62	M	13.7	Never		47.9	195.6	60.9	+	+		+	7.1	5.3	+	0.23
12	65	F	17.9	Never		75.4	144.9	123.9	+			+	8.9	6.8	+	0.47
13	62	M	20	33		74.1	106.9	89.3	+			+	8.0	5.2	+	0.43
14	62	M	22	Never		52.8	111.4	62.5	+			+	13.3	8.8		0.79
15	71	F	13.3	Never		37.7	179.3	ND	+			+	8.6	6.5	+	0.25
16	52	F	15.2	Never		60.4	183.1	76.5					10.9	7.8		0.34
17	70	M	16.5	32		43.6	153.2	38.5		+		+	5.4	3.7	+	0.20
Total (number)		11;6		7	1				12	4	2	14			9	
Average	66.6		17.8			65	142	85						8.1		0.44
IPF																
1	75	F	22.4	Never		81.4	98.1	51.2					15.1	11.7		0.65
2	75	M	26.5	30		99.1	105.1	58.3					10.2	7.0		0.79
3	79	M	28.2	14		85.9	91	71.6					21.5	14.7		1.07
4	68	M	26.6	33		119.5	50.2	75.9					13.4	9.0		0.38
5	43	M	23.1	15	+	74.6	89.1	62.4					16.9	11.8		0.79
6	79	F	24.8	25		96.7	91.6	53.6					18.3	14.3		1.17
7	61	M	22.2	Never		100.6	84.5	87.8					4.1	2.6	+	0.88
8	84	M	20.7	25		77.2	64.3	62.1					9.7	7.2		0.57
9	70	M	25.4	66		60.1	76.3	69.7					14.2	10.4		1.07
10	83	M	25.8	33		95.4	84.9	88					12.6	10.2		0.74
FHP																
1	58	F	26.7	0.3		73.3	125.7	55.8					21.3	13.5		0.51
2	50	F	22.9	32		67.5	114.6	68.3					11.5	8.6		0.45
3	78	M	17.1	Never		64.7	87.8	71.9					10.6	7.3		0.85
4	68	M	23.4	18		100	89.1	86					13.5	8.5		0.86
5	61	M	24	63	+	104.1	73.7	75.6					24.9	15.4		0.96
Total (number)		11; 4		12	2				0	0	0	0			1	
Average	68.8		24.0			86.7	88.4	69.2						10.1		0.78

*Abbreviations*: *BMI* body mass index, *CVJ* costovertebral joint, *DLco* diffusing capacity of the lung for carbon monoxide, *FHP* fibrotic hypersensitivity pneumonitis, *IPPFE* idiopathic pleuroparenchymal fibroelastosis, *IPF* idiopathic pulmonary fibrosis, *LVCR* lung volume change ratio, *MSJ* manubriosternal joints, *Pred* predicted, *RV* residual volume, *SCJ* sternocostal joint, *TLC* total lung capacity, *TMR* thoracic motion ratio, *VC* vital capacity

**Table 2:** Comparison between thoracic motion, lung volume change, and PFT parameters in IPPFE and non-PPFE groups

	PPFE (*n* = 17)	Non-PPFE (*n* = 15)	*p*-value
TMR	6.80 [5.00–8.60]	10.20 [7.90–12.65]	0.041
LVCR	0.43 [0.23–0.48]	0.79 [0.61–0.92]	< 0.001
VC (%pred)	68.30 [47.90–74.10]	85.90 [73.95–99.55]	0.007
RV/TLC (%pred)	133.00 [114.70–179.30]	89.10 [80.40–94.85]	< 0.001
DLco (%pred)	87.60 [72.55–102.45]	69.70 [60.20–75.75]	0.023

*Abbreviations*: *DLco* diffusing capacity of the lung for carbon monoxide, *LVCR* lung volume change ratio, *Pred* predicted, *RV* residual volume, *TLC* total lung capacity, *TMR* thoracic motion ratio, *VC* vital capacity

## Thoracic joint abnormalities

Abnormalities of thoracic joints were observed in 14 of 17 IPPFE patients (82%), while none were observed in the non-PPFE patients (Table 1).

1. SCJs.
  SCJ abnormality was found in 12/17 (71%) patients with IPPFE and in 0/15 patients without PPFE. Figure 2A shows normal SCJs in a case of fHP (#FHP2). Although mild ossification of the costal cartilages is present, no calcification is observed in the joint spaces. Figure 2B shows calcified SCJs in the bilateral third, fourth, and fifth ribs in an IPPFE case (#IPPFE3), where calcified SCJ ligaments bridge the sternal body and the costal cartilages. As shown in Fig. 1, #IPPFE1 and #IPPFE2 also had six calcified SCJs. Figure 2C shows severe calcification of the SCJs with ossification of the costal cartilages, in which the thoracic cage appears like an immovable armor (#IPPFE4).
  **Fig. 2:**
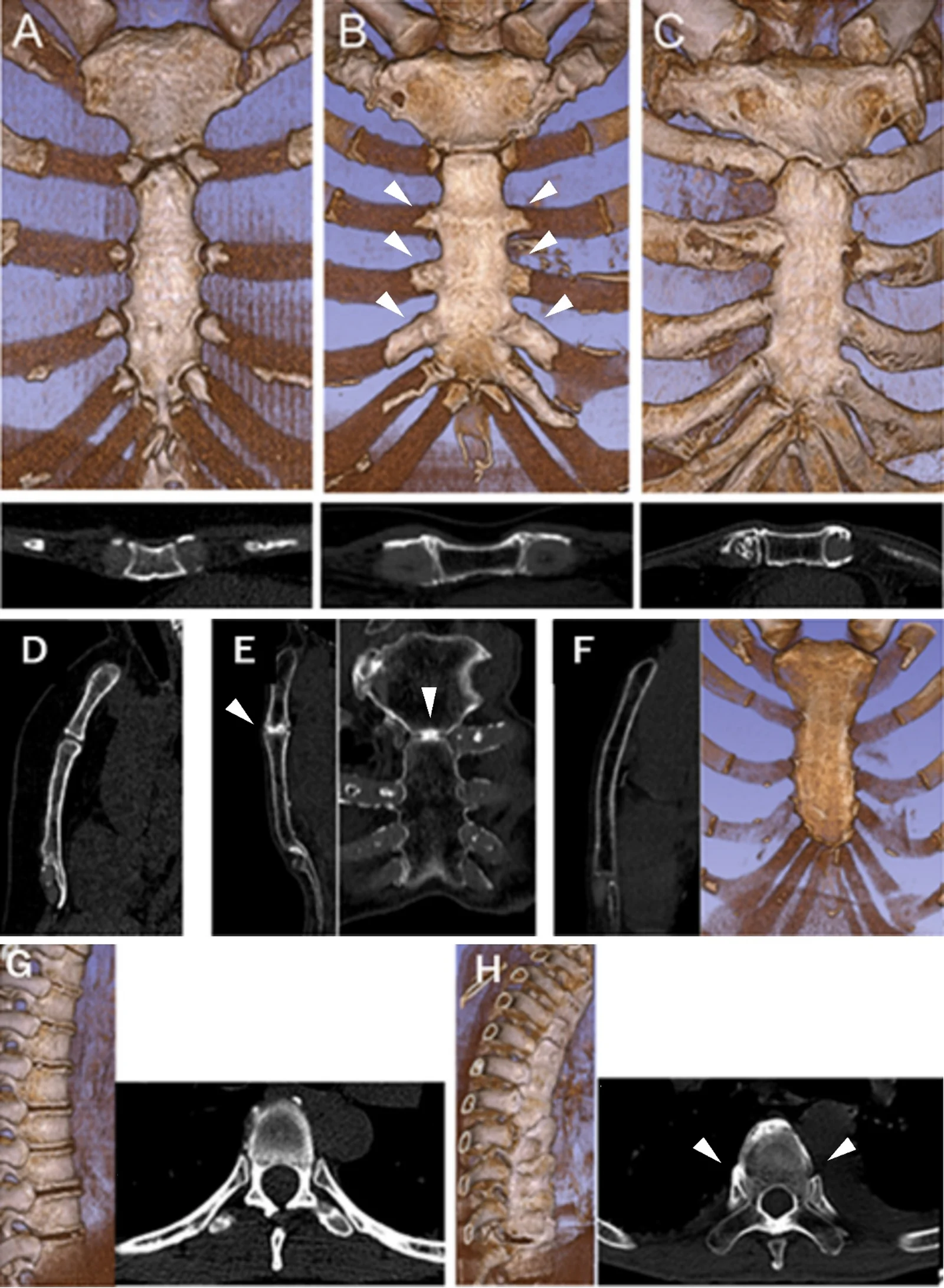
Representative cases of thoracic joint abnormalities in IPPFE and non‑PPFE ILDs. **A** Normal sternocostal joints (SCJs) in a case of fibrotic hypersensitivity pneumonitis (fHP; #FHP2). Mild ossification of the costal cartilage was observed; however, the SCJs remained intact. **B** Calcified SCJs of the third to fifth ribs in an IPPFE case (#IPPFE3) (white arrowhead), where calcified ligaments bridge the sternal body and costal cartilages. **C** Extensive calcification of the SCJs with ossified costal cartilages in an IPPFE case (#IPPFE4), resulting in a thoracic cage resembling an immobile armor. Frontal view by volume rendering (upper) and horizontal CT image at the fifth SCJ with the bone window setting (lower) are shown. **D** Normal manubriosternal joint (MSJ) in an fHP case (#FHP2). **E** Calcification of the MSJ in an IPPFE case (#IPPFE2) (white arrowhead). **F** Complete fusion of the MSJ with severe asymmetry of the 2nd–4th SCJs and cartilages in an IPPFE case (#IPPFE5). **G** Normal costovertebral joints (CVJs) in an fHP case (#FHP2). **H** Abnormal CVJs in an IPPFE case (#IPPFE4), showing marked vertebral deformity and adhesion of thoracic vertebral column (left) and the sixth CVJ on axial view (right). Thoracic spine deformity and joint fusion due to the loss of articular space and calcification are noted (white arrowhead)
2. MSJ
  MSJ abnormality was found in 4/17 (24%) patients with IPPFE and in 0/15 patients without PPFE. Figure 2D shows a normal MSJ in a case of fHP (#FHP2). Figure 2E shows a calcified MSJ in an IPPFE case (#IPPFE2). Complete, non-calcified fusion between the manubrium and the body was found in 4/17 (24%) patients with IPPFE and 3/15 (20%) patients without PPFE, a frequency similar to that reported in Ref. 17. Of these, an IPPFE case (#IPPFE5), shown in Fig. 2F, exhibited severe asymmetry of the SCJ positions and costal cartilage orientation, which may cause abnormal thoracic motion. Therefore, we judged this case as having an abnormal SCJ.
3. CVJs
  CVJ abnormality was found in 2/17 (12%) patients with IPPFE and in 0/15 patients without PPFE. Figure 2G shows normal CVJs (**#**FHP2), and Fig. 2H shows abnormal CVJs (#IPPFE4), in which marked deformation of the thoracic vertebral column and a horizontal cross-sectional view of the fused sixth CVJ are observed.

### Thoracic motion analyses

**Fig. 3:**
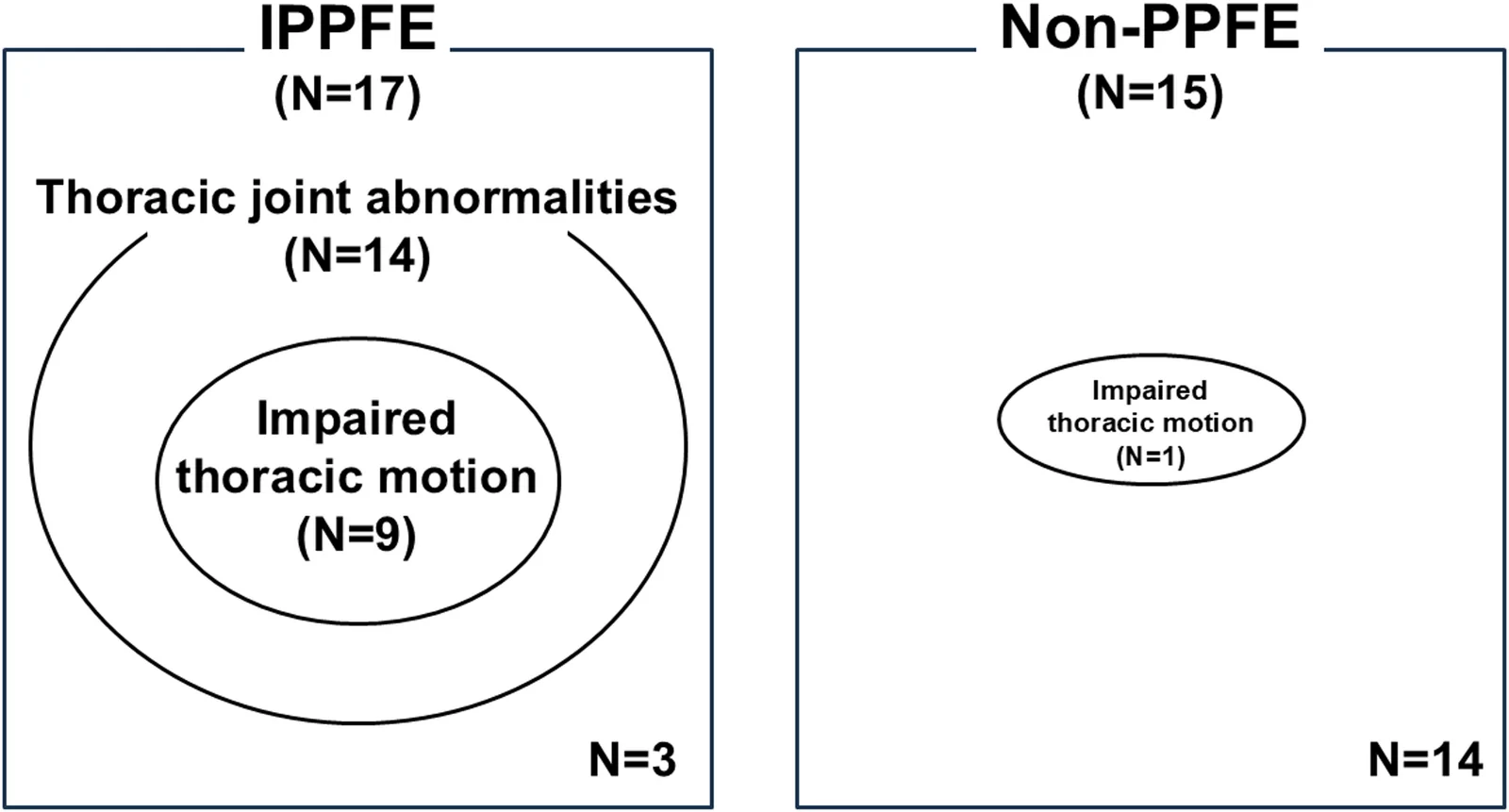
Venn diagram summarizing the findings in IPPFE. Among the 17 patients, 9 exhibited impaired thoracic motion, all of whom exhibited thoracic joint abnormalities. Impaired thoracic motion was identified in only 1 of the 15 non-PPFE patients. Abnormalities of the thoracic joints were observed in 14 of the 17 IPPFE patients, whereas none were observed in the non-PPFE patients

The absolute values of the thoracic moving distance, the TMR, and the LVC between inspiratory and expiratory images are shown in Table 1. Compared to the non-PPFE group, the PPFE group exhibited significantly lower median values for TMR (6.80 [5.00–8.60] vs. 10.20 [7.90–12.65], *p* = 0.041) and LVCR (0.43 [0.23–0.48] vs. 0.79 [0.61–0.92], *p* < 0.001) (Table 2). Impaired thoracic motion, defined as < 7% of TMR, was identified in 9 of 17 IPPFE cases (53%), but only in 1 of 15 non-PPFE cases (7%). All IPPFE cases with impaired thoracic motion had thoracic joint abnormalities (Table 1; summarized and depicted in Fig. 3). This one case (#IPF 7) exhibited paradoxical movement (downward and posterior displacement) at the sixth rib, and the thoracic moving distance was calculated without the sixth rib data. Except for this case, all sites moved upward and anteriorly, and no relationship was observed between the motion and the lung lesions.

### Associations between thoracic motion, lung volume change, and pulmonary function test (PFT) parameters

**Fig. 4:**
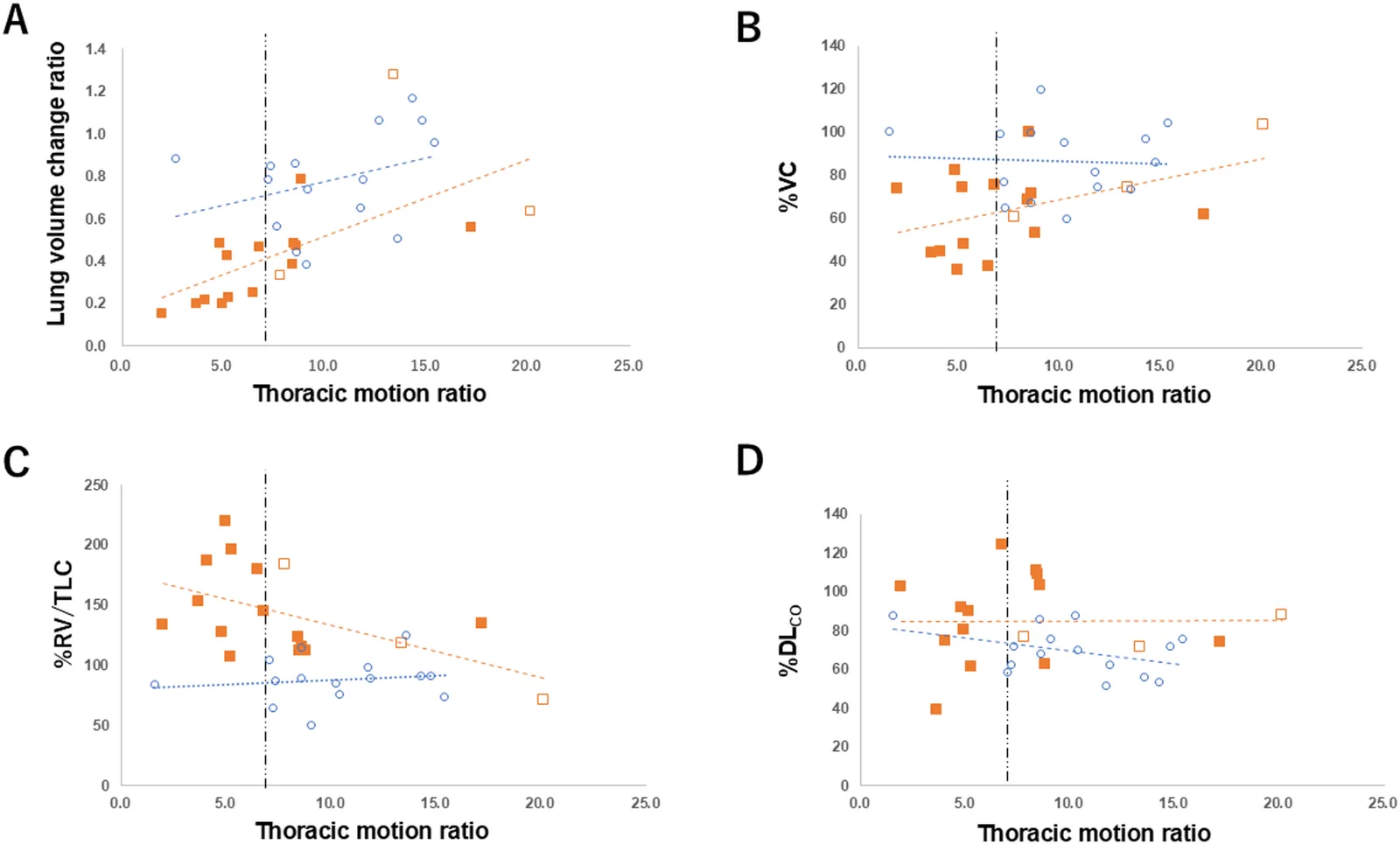
Scatter plots with linear regression lines illustrating the associations of TMR with LVCR and PFT parameters in the IPPFE and non-PPFE groups: **A** TMR and LVCR, (**B**) TMR and %VC, (**C**) TMR and %RV/TLC, and (**D**) TMRand %DLco. Square and circle markers represent IPPFE and non-PPFE cases, respectively. Closed and open markers indicate cases with and without joint abnormalities, respectively. Dotted black lines denote the threshold for impaired thoracic motion. LVCR was calculated from paired expiratory and inspiratory CT images. Orange and blue dotted lines indicate linear regression lines for the IPPFE and non-PPFE groups, respectively. DLco, diffusing capacity of the lung for carbon monoxide; IPPFE, idiopathic pleuroparenchymal fibroelastosis; LVCR, lung volume change rate; PFT, pulmonary function test; RV, residual volume; TLC, total lung capacity; TMR, thoracic motion ratio; VC, vital capacity

We investigated the associations between TMR and LVCR, as well as PFT parameters, including %VC, %RV/TLC, and %DLco. In the IPPFE group, particularly among patients with joint abnormalities, reduced TMR and LVCR values were frequently observed. The correlation between TMR and LVCR tended to be stronger in the IPPFE group (*r* = 0.829, *p* < 0.001) than in the non-PPFE group (*r* = 0.304, *p* = 0.27) (Fig. 4A). Most patients with IPPFE and joint abnormalities exhibited reduced %VC and increased %RV/TLC (Fig. 4B, C). The correlation of TMR with %VC and %RV/TLC tended to be stronger in the IPPFE group ( *r* = -0.529, *p* = 0.031 and *r* = -0.036, *p* = 0.903, respectively) than in the non-PPFE group (*r* = 0.353, *p* = 0.165 and *r* = 0.157, *p* = 0.576, respectively), whereas the association with %DLco was negligible in the IPPFE group ( *r* =-0.018, *p* = 0.954) (Fig. 4B–D). These findings suggest that thoracic joint abnormalities and impaired thoracic motion may be characteristic features associated with IPPFE.

## Discussion

This study provided novel evidence that joint abnormalities and impaired motion of the thoracic cage are more frequently observed in patients with IPPFE than in patients without PPFE. Thoracic motion was more associated with lung volume change in the IPPFE group. All patients with IPPFE with impaired thoracic motion demonstrated thoracic joint abnormalities, implying their potential relationship. These findings imply the role of structural thoracic alterations in the pathophysiology of impaired thoracic motion and potentially in the development of IPPFE.

Anteroposterior flattening of the chest (flat chest or platythorax) is a hallmark of IPPFE [20]. Ikeda et al. suggested a causal relationship between the progression of fibroelastosis and that of platythorax in patients with PPFE [21]. However, it is also possible that both conditions are influenced by a common confounding factor. Our findings raise the possibility that anterior thoracic restriction due to thoracic joint immobility may contribute to both platythorax and fibroelastosis in the upper lobes. In the upright or supine position, limited anterior chest expansion may preferentially impair ventilation in gravity-independent regions, such as the upper or anterior lung fields. It is well known that hypoventilated parenchyma is prone to atelectasis.

Another important potential confounder is the distribution of fibrosis. Upper-lobe fibrosis itself may mechanically restrict thoracic expansion and reduce TMR. Although we included fHP as a comparator with relative upper-lobe involvement, the small sample size precluded formal matching by fibrosis location or extent. Therefore, the observed findings should not be considered entirely specific to IPPFE. Future matched studies are needed.

According to the ERS/ATS guidelines [22], restrictive impairment on PFTs is defined as a reduction in lung volume that may reflect intrinsic parenchymal factors or an inability to fully inhale due to extrapulmonary factors (e.g., respiratory muscle weakness, chest wall abnormalities, or obesity). In other words, a decrease in %VC may be attributable to chest wall abnormalities. The ERS/ATS guidelines also state that an increase in RV/TLC may result from lung hyperinflation or complex restriction [22]. Complex restriction is associated with processes that impair lung emptying, such as chest wall restriction [22, 23]. In contrast, DLco reflects the gas transfer capacity of the lung parenchyma and is generally independent of chest wall motion [22]. When these three parameters are considered together, the primary mechanisms underlying different patterns of ventilatory impairment may be summarized as follows. Pulmonary fibrosis, characterized by low VC, normal RV/TLC, and low DLco, is mainly caused by impaired parenchymal expansion. Pulmonary emphysema, characterized by normal VC, elevated RV/TLC, and low DLco, is primarily caused by impaired parenchymal emptying. PPFE, characterized by low VC, elevated RV/TLC, and preserved DLco, may be associated with chest wall abnormalities.

**Fig. 5:**
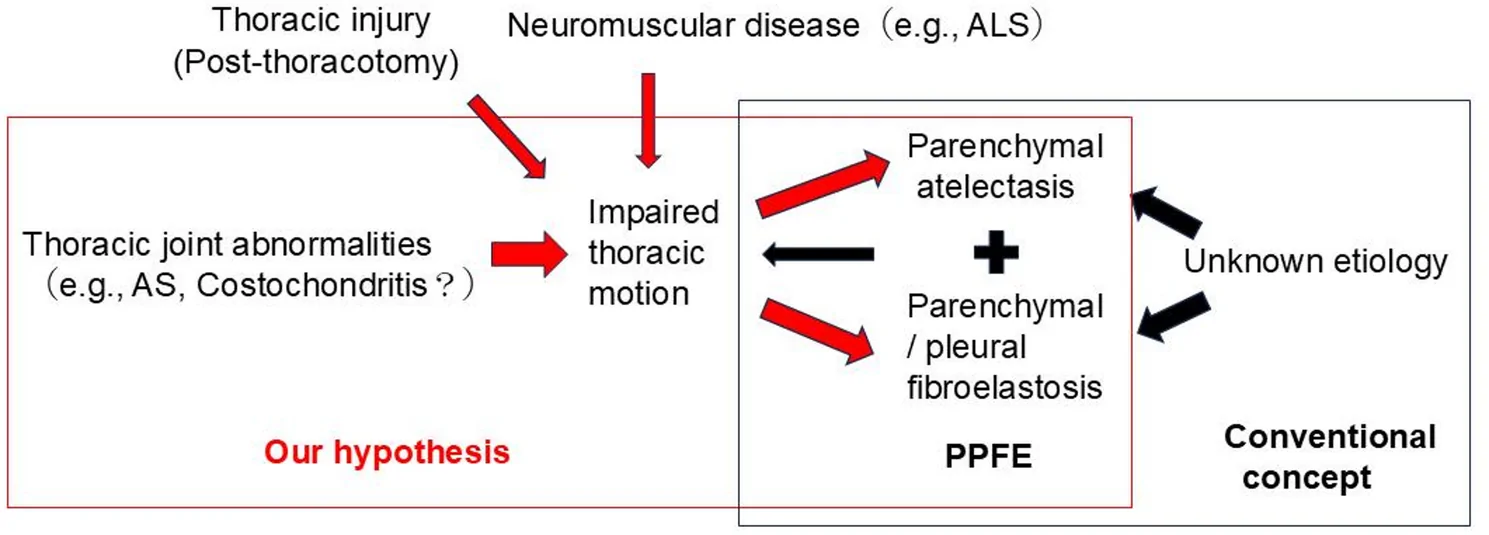
Proposed conceptual framework for the pathogenesis of PPFE. Our hypothesis is indicated by red arrows with red squares, whereas the conventional concept is indicated by black arrows with black squares. Note that the arrows between impaired thoracic motion and pleuroparenchymal lesions point in opposite directions. AS, ankylosing spondylitis; ALS, amyotrophic lateral sclerosis

Our findings regarding the correlations between TMR and PFT parameters demonstrated that, in IPPFE, TMR was significantly associated with ventilatory parameters (VC and RV/TLC), but not with gas transfer capacity (DLco). These findings support the above hypothesis that IPPFE is caused by chest wall abnormalities. The joint abnormalities observed in this study are unlikely to be secondary to long-standing immobility, because calcific changes secondary to contracture are not reported in the literature [24, 25]. We therefore propose a novel conceptual schema for PPFE (Fig. 5), in which the conventional concept and our hypothesis are indicated by black and red arrows, respectively.

Costochondritis, historically termed Tietze’s syndrome, is a benign, nonsuppurative inflammatory condition characterized by localized swelling and pain of the SCJs and MSJs, with symptoms typically resolving spontaneously without specific treatment [26]. Beyond costochondritis, enthesitis associated with autoimmune diseases can occur in the joints of the thoracic cage, even in the absence of a specific diagnosis. Although no patients in this study had a documented history of costochondritis, it is possible that symptoms were too mild to be recognized or recalled and that joint calcifications may have remained as sequelae. In addition, particularly in an elderly population, degenerative joint disorders and age-related musculoskeletal changes may contribute to thoracic joint abnormalities.

Elastofibroma is a rare benign soft-tissue tumor that typically occurs in the infrascapular region between the scapula and the thoracic wall, most frequently in elderly individuals engaged in heavy labor [27, 28]. Although the pathogenesis of this lesion remains unclear, it has been proposed that repetitive microtrauma caused by friction between the scapula and the thoracic wall induces reactive hyperproliferation of fibroelastic tissue [28]. This hypothetical mechanism may also be applicable to pleural thickening in PPFE. Because the visceral and parietal pleurae slide against each other during respiration, impaired thoracic motion may lead to repetitive abnormal friction and consequently contribute to pleural fibroelastosis.

This study had some limitations. First, it has a single-center retrospective design and small sample size, which reflects the rarity of IPPFE. Second, expiratory CT is not routinely performed in PPFE evaluation, potentially introducing a selection bias. Third, quantitative methods for evaluating joint abnormalities have not yet been established. Image assessments were conducted through consensus among the investigators, and formal inter- and intraobserver agreement analyses were not available. Fourth, longitudinal changes in thoracic joint abnormalities and thoracic motion were not assessed. To clarify the disease-specific significance of the observed abnormalities, large-scale longitudinal studies, including an age-, sex-, and BMI-matched healthy control group, as well as disease control groups matched for fibrosis distribution and extent, are required. Finally, a definitive causal relationship among thoracic joint abnormalities, mobility, and pulmonary lesions remains to be established.

## Conclusions

Thoracic joint abnormalities associated with impaired motion are frequently observed in IPPFE patients. These abnormalities may restrict thoracic movement and lead to the development of IPPFE.

## Data Availability

The datasets used and/or analyzed during the current study are available from the corresponding author on reasonable request.

## Abbreviations

ATS: American Thoracic Society
BMI: Body mass index
CVJ: Costovertebral joint
DLco: Diffusing capacity of the lung for carbon monoxide
ERS: European Respiratory Society
fHP: Iibrotic hypersensitivity pneumonitis
IPPFE: Idiopathic pleuroparenchymal fibroelastosis
IPF: Idiopathic pulmonary fibrosis
ILD: Interstitial lung disease
LVCR: Lung volume change ratio
MDCT: Multidetector computed tomography
MSJ: Manubriosternal joints
Pred: Predicted
PFT: Pulmonary function test
RV: Residual volume
SCJ: Sternocostal joint
3D-CT: Three-dimensional computed tomography, TLC, total lung capacity
TMR: Thoracic motion ratio
VC: Vital capacity
